# Analyzing the application of mixed method methodology in medical education: a qualitative study

**DOI:** 10.1186/s12909-024-05242-3

**Published:** 2024-03-04

**Authors:** Abdulaziz Ibrahim Alhassan

**Affiliations:** 1 Department of Medical Education, College of Medicine, King Saud bin Abdulaziz University for Health Sciences, Riyadh, Saudi Arabia; 2 King Abdullah International Medical Research Center, Riyadh, Saudi Arabia; 3 College of Medicine, Department of Medical Education King Saud bin, Abdulaziz University for Health Sciences (KSAU-HS), KSAU-HS- Riyadh, 11481, +966-11- 4299999 Riyadh, P.O. Box 3660, Saudi Arabia

**Keywords:** Mixed methods methodology, Medical education research, Medicine

## Abstract

**Background:**

Interest in mixed methods methodology within medical education research has seen a notable increase in the past two decades, yet its utilization remains less prominent compared to quantitative methods. This study aimed to investigate the application and integration of mixed methods methodology in medical education research, with a specific focus on researchers’ perceptions, strategies, and readiness, including the necessary skills and expertise. This study adheres to the COREQ guidelines for reporting qualitative research.

**Methods:**

Faculty members from King Saud bin Abdulaziz University for Health Sciences (KSAU-HS), Saudi Arabia, across its three campuses in Riyadh, Jeddah, and Al Ahsa, participated in this study during the 2021–2022 academic year. We conducted 15 in-depth, one-on-one interviews with researchers who had previously used mixed methods in their medical education research. Theoretical saturation was reached with no refusals or dropouts. Data were collected using a semi-structured interview guide developed from literature review and mixed methods guidelines. Thematic analysis was employed to analyze the data, enabling a comprehensive understanding of the participants’ perspectives.

**Results:**

The thematic analysis of the interviews yielded three key themes. The first theme, ‘Understanding and Perceptions of Mixed Methods in Medical Education Research,’ delved into researchers’ depth of knowledge and conceptualization of mixed methods. The second theme, ‘Strategies and Integration in Mixed Methods Implementation,’ explored how these methodologies are applied and the challenges involved in their integration. The final theme, ‘Mastery in Mixed Methods: Prerequisites and Expert Consultation in Research,’ highlighted the gaps in readiness and expertise among researchers, emphasizing the importance of expert guidance in this field.

**Conclusion:**

Findings indicate a varied understanding of mixed methods among participants. Some lacked a comprehensive grasp of its application, while others perceived mixed methods primarily as a means to enhance the publication prospects of their studies. There was a general lack of recognition of mixed methods as a guiding methodology for all study aspects, pointing to the need for more in-depth training and resources in this area.

## Background

The mixed methods methodology has been used within the social sciences for nearly 100 years. As early as 1938, mixed-methods methodology was used in an anthropological study conducted in Southeast Asia [1]. The use of mixed methods has become strongly engrained within the social sciences as students are routinely educated about mixed methods methodology and various numbers of textbooks include discussions and guidelines about how to conduct mixed methods research [2]. In medicine and medical education, the mixed methods methodology is relatively new with attention being given to the methodology only within the past 20 years [3]. Even with a growing interest in mixed methods research within medicine, the use of this methodology has been met with on-going debate about its usefulness, how it should be used, and whether it is a distinct research methodology or whether it is just a combination of qualitative and quantitative research methods [4].

The reason that mixed methods is still relatively new and not heavily utilized within medicine may be because this methodology requires a broader range of skills and more time and effort to carry out as compared to quantitative studies [5]. In addition, medical researchers and practitioners have historically believed that quantitative research and a reliance on objective results and findings is more appropriate than qualitative measures that are perceived to be based on purely subjective analysis [6]. The idea that useful information and findings are only available from statistical analysis has likely led to the idea that mixed methods do not provide more useful data and a broader understanding of issues and problems.

If the desire exists to increase the understanding and use of mixed methods methodology in medicine and medical research, then it is necessary to understand the level of awareness and knowledge that exists about mixed methods among medical education researchers. If medical education researchers do not understand mixed methods methodology, then they are less likely to use it. However, medical education researchers may understand mixed methods methodology, but view it as being inferior to quantitative methods. The purpose of this study is to examine the use of mixed method methodology in medical education research, the level of awareness about the reason to use mixed method methodology, how it is utilized, and its characteristics. By examining the knowledge that medical education researchers possess about mixed methods methodology and how they use the methodology, it may be possible to better understand where gaps in knowledge exist, and, in turn, increase the use of mixed methods methodology among medical education researchers. The importance of this study is that rather than examining why mixed methods methodology is still used less in medicine than the social sciences, the focus is on what medical education researchers know about mixed methods. With the information gained from this study, it will be possible to better understand the actions that may need to be taken in order for medical education researchers to more actively use mixed methods methodology in medical education.

## Defining mixed methods methodology

It may seem unnecessary to define mixed methods methodology for researchers who are already interested, and likely knowledgeable, about this research methodology. However, in a study in which the goal is to understand the knowledge that exists among medical education researchers about mixed methods methodology, it is appropriate to briefly discuss the different definitions that exist. Qualitative and quantitative research methods are often discussed as being in opposition to each other with quantitative research being used to test hypotheses and qualitative research being used to investigate the lived experiences and social interactions of individuals and groups [7].

From a broad perspective, mixed methods methodology has been defined as a research methodology that combines elements of qualitative and quantitative methods to gain more depth and understanding of a problem or topic [8]. However, Creswell & Plano Clark (2011) explained that the mixed methods methodology is more than just combining qualitative and quantitative methods [9]. Instead, mixed methods is the use of both qualitative and quantitative methods in a single study in a way that builds on each other to answer a given research question. In this regard, mixed methods methodology is not about using 50% qualitative and 50% quantitative methods in a single study. Instead, mixed methods methodology involves the use of qualitative and quantitative methods based on what is needed and appropriate to answer a research question.

Mixed methods methodology has also been argued to be distinct and separate from other research methods [10]. Rather than solely being a combination of qualitative and quantitative methods, mixed methods methodology has its own vocabulary and techniques with the goal of not only answering research questions, but also applying scientific findings to actual practice [9]. The mixed methods methodology is a distinct third research methodology separate from qualitative and quantitative methodology that involves the integration of quantitative and qualitative data in order to gain a more in-depth understanding of problems and issues [11]. In this regard, the mixed methods methodology extends beyond discovering whether variables are related to each other or whether certain conditions are found to exist in relation to specific phenomena. Instead, mixed methods is about taking the results of scientific study and applying them to practice, such as applying the results of medical studies to medical practice.

## Why is mixed methods limited in medical research

There are several reasons for the limited use of the mixed methods methodology within medicine and medical research. These reasons are noted in Table 1. One of the reasons for the limited use of mixed methods in medicine is because medical research has traditionally been focused on objective findings and the idea that there should be a separation between objective factors and subjective feelings and opinions [4]. Quantitative research has often been viewed as being related to a single truth that can be discovered by analyzing numerical data while qualitative research involves many truths based on the perceptions and ideas of those who are being studied [12]. The idea that qualitative research methods yield many truths has been viewed as inappropriate or not specific enough within the realm of medicine.

**Table 1:** Why the Use of Mixed Methods Methodology is Limited in Medical Research

• 0048istorical reliance on quantitative findings in medicine
• Greater complexity in conducting mixed methods research
• Lack of instructions in medical education about mixed methods methodology

In addition, conducting mixed methods research is more complex than conducting quantitative research [5]. Sawatsky et al. (2019) argued that even with advanced training in mixed methods methodology, it can be difficult for medical clinicians to understand and engage in mixed methods research [13]. The result of the difficulty and lack of understanding in conducting mixed methods research can lead to medical research in which sociologists and other social scientists conducting the qualitative portion of a mixed methods study and medical researchers conducting the quantitative portion [14].

Curry et al. (2013) also argued that another reason for the lack of mixed methods research in medicine is because of the lack of information and instruction about how to conduct mixed methods studies within journals pertaining to biomedical and health services [15]. The lack of information and guidance provided to medical researchers has resulted in a lack of use of mixed methods methodology.

## Benefits of mixed methods research in medicine

A question that might arise is why mixed methods research is important or useful within medical research. If medical research is about finding the connections between variables or discovering why certain conditions might be related to specific outcomes, then quantitative research might be most appropriate. One of the arguments that has been made about the importance of mixed methods methodology in medical research is the ability to expand the results of scientific studies so they can be used in actual practice [16]. Mixed method studies can provide information and insights that lead to more robust assessments and practice and increased competency within medical education than is possible through quantitative research alone [6].

Thistlehwaite et al. (2012) argued that quantitative studies are no longer enough in terms of evidence-based practice for medical education [17]. Instead, the idea of evidence must be expanded for the benefit of both teaching medical information and findings and engaging in the process of learning medical information. The combination of quantitative and qualitative findings can lead medical practitioners to better implement interventions to patients that consider the social issues that influence patient actions and interactions with medicine and medical conditions [18]. In this regard, the use of mixed methods methodology can allow practitioners to use medical research in actual practice to improve patient care. Glasgow et al. (2012) argued that even with the advantages of methodological approaches such as mixed methods methodology, future healthcare practitioners and scientists are not being trained in how to use those methodologies to impact actual practice [19].

### Mixed methods in medical education

While mixed methods have been gradually gaining traction in medical research, their application in medical education is distinct yet equally vital. Medical education encompasses a broad range of areas including evaluation, assessment, faculty development, curriculum design, and teaching methods. Mixed methods research offers a nuanced approach to understanding these complex and multifaceted aspects. For instance, it allows for a comprehensive evaluation of educational interventions by quantitatively measuring their outcomes while qualitatively exploring the experiences and perceptions of learners and educators [20]. This dual approach is essential in a field where both measurable outcomes and subjective experiences are crucial.

### Bridging quantitative and qualitative paradigms in education

In medical education, the integration of mixed methods can bridge the gap between quantitative assessments of educational efficacy and qualitative insights into educational experiences. This integration is particularly important in areas like curriculum development and faculty training, where understanding both the effectiveness and the experiential aspects can lead to more informed and holistic improvements [21].

### Identification of the gap

#### The gap in mixed methods utilization

Despite the potential benefits, there’s a noticeable gap in the effective application of mixed methods in medical education. A significant portion of medical education researchers comes from a background strongly rooted in quantitative methods, often leading to a preference for, or over-reliance on, these methods. The challenge arises when researchers, familiar with qualitative and quantitative methods individually, attempt to conduct mixed methods research without a comprehensive understanding of its unique nature [20].

#### Misconceptions and challenges

The misconception that familiarity with both qualitative and quantitative methods automatically equates to proficiency in mixed methods can lead to suboptimal research designs. Mixed methods research is not simply a juxtaposition of two methodologies; it requires an understanding of how to blend these approaches cohesively, with its own strategies, theoretical frameworks, and specific types of research questions [22]. Researchers might utilize mixed methods due to the availability of both data types or under the mistaken belief that it inherently enhances the study, without a clear rationale for its use. This can result in poorly integrated results where quantitative and qualitative findings are reported separately without meaningful synthesis [22].

## Study objective

This study aims to investigate the application and integration of mixed methods methodology in medical education research. It focuses on understanding researchers’ perceptions, their strategies in applying mixed methods, and their readiness in terms of skills and expertise required for effective implementation. The objective is to uncover how mixed methods are currently being used in medical education research, identify the gaps in knowledge and application, and provide insights into how these gaps can be bridged for more robust and effective educational research.

## Methodology

### Study design

A qualitative research method utilizing one-on-one in-depth interviews with the researchers, who previously used mixed methods research as part of their medical educational research, was used to achieve the objectives of the study. A qualitative approach was considered appropriate specifically phenomenology, to investigate the application and integration of mixed methods methodology in medical education research and to fully understand the reasons and rationale of using a mixed methods approach by the study participants. In this study, it allowed for an in-depth exploration of how researchers in medical education perceive, experience, and interpret mixed methods methodology. By focusing on their personal narratives, phenomenology provided a framework for understanding the complexities and nuances in their perspectives, which is central to addressing the research objectives.The interviews were conducted through the main author (AA), utilizing a constructed semi-structured interview guide composed of open-ended questions formulated after a review of the literature and considering the guidelines for using appropriate mixed method methodology.

### Setting

The study was conducted at King Saud bin Abdulaziz University for Health Sciences (KSAU-HS), Saudi Arabia in 2021–2022 academic year. The university has three campuses situated in three different cities, Riyadh, Jeddah, and Al Ahsa. The university provides both undergraduate and postgraduate programs. The university requires the students to complete research projects within their educational programs supervised by faculty members. The university also encourages and supports its faculty members to conduct their own research as part of their expected academic roles.

### Study population

Inclusion criteria: all researchers who had conducted mixed method methodology research, including faculty members and postgraduate students were included.

Exclusion Criteria: any researcher who had conducted mixed methods research that was not in the field of medicine nor medical education was excluded.

### Sample size

Considering that the nature of utilizing mixed methods methodology is not very common compared to using quantitative or qualitative studies alone, an initial sample size considered to generate sufficient data was between six and twelve researchers. However, the sample size was guided by theoretical saturation which was reached after 15 participants with no refusal nor drop outs during the study.

### Sampling technique

A purposive sampling technique was used. The researcher began interviewing faculty members in the field of medical education who taught postgraduate medical educational students, graduate students in the medical education program, and any university faculty member.

### Data collection

The interview guide included a series of questions developed based on a literature review to analyze and determine the proper utilization and application of the mixed method methodology design. The researcher followed the interview guide, consisting of 15 open-ended questions. The guide consisted of questions on demographic data such as gender, years of experience, researcher background, academic standing, and whether the participants supervised any students in case they were faculty members. The interview guide was piloted by five expert members in medical education to ensure clarity and coherence of the questions.

All participants were asked to sign a consent form, and the researcher explained the aims of the study, the value of their input to the study, and that their participation was voluntary, and that they had the right to withdraw from the interview at any time during the data collection. The interviews were conducted face-to-face in English and audio recorded for quality purposes of data collection and analysis. The interviews lasted from 40 to 60 min in duration. For validity and credibility, the data was checked by two expert members. Once data saturation was obtained, the interviews were discontinued. The transcripts were returned to participants for comments and concretion.

### Data analysis

The interviews were imported in NVIVO (QSR international) version 12 for analysis. AA transcribed the interviews verbatim, and AA verified the interview text in consultation with the expert member check NA. A thematic analysis was utilized in which the interview text was read multiple times to become familiar with the content of the data, and then the most relevant and appropriate codes were assigned by the coders AA and NA, summarizing the meaning of the participant expressions in phrases and lines. This was followed by logical editing of code names. Following this, the codes explaining a common pattern in the data were aligned under candidate themes. The themes were finalized, and the coded data with code names and participant references was exported to a word document. The themes were finalized in consultation with AA and the text were read again for potential inconsistencies in coding or theme assignment before interpretation and editing were performed to summarize the findings from the analysis.

## Reflexivity

In conducting this research, I, Abdulaziz Alhassan, have been mindful of my positionality as an assistant professor with expertise in mixed methods methodology, which might influence the research process and interpretation of findings. My experience in chairing evaluation and educational program units and teaching postgraduate courses at KSAU-HS has both informed my understanding and necessitated a conscious effort to maintain objectivity.

To ensure the trustworthiness of the research, I employed several strategies. Firstly, triangulation was used by combining different data sources and perspectives, interviewing a diverse range of principal investigators at KSAU-HS. This approach adds depth and breadth to the findings, ensuring that they are not solely reflective of my interpretations. Secondly, member checking was conducted, where findings were shared with some participants for validation. This process ensured that the interpretations accurately reflected their experiences and perceptions, thereby maintaining the integrity of the data. Lastly, reflective journaling played a crucial role throughout the study. I maintained a research journal to document personal reflections, potential biases, and decisions made during the research process. This practice was instrumental in continuously evaluating and mitigating any personal biases that might have influenced the study.

The research was conducted in an environment familiar to me– KSAU-HS. While this familiarity provided a rich contextual understanding, it also required careful attention to avoid bias. My interactions with participants were conducted professionally, with an emphasis on eliciting their views and experiences without imposing my own perspectives.

By acknowledging my background and actively engaging in reflexivity, I aimed to enhance the credibility and reliability of the research while being transparent about my influence on the study.

## Findings

Our analysis identified three themes from the data which were distinct and unique, explaining the construct of the perceptions of using mixed methods in research related to medical education. These themes included:: understanding and perceptions of mixed methods in medical education research, strategies and integration in mixed methods implementation, and mastery in mixed methods: prerequisites and expert consultation in research. No modifications were provided by the participants’ feedback on the findings.

### Theme 1: understanding and perceptions of mixed methods in medical education research

The participants offered a range of definitions, associating them with what they believed was comprised of mixed methods research. A few participants believed that the presence of quantitative and qualitative methods in a single study comprised of a mixed methods design. Most believed that it was a design of research that merges a quantitative part with a qualitative part. Others believed that since it is comprised of two components of quantitative and qualitative research, the qualitative research is an inductive part of the research while quantitative research is more of a deductive mode of inquiry; whereas the data that originates from both types of research is called mixed methods design. Some participants believed that the mixing of data during the analytic part consisted of mixed methods research. Most had a clear understanding of the composition of mixed methods research.

*Mixed methods is the presence of quantitative and qualitative studies in a single study. 5-Female*.

*Usually, it involves mixing quantitative and qualitative methodologies in one research project. 12-Female*.

*It is a combination of exploratory and explanatory methods. It is transferring one piece of data to another or merging data together. 9-Male*.

*It’s when we analyze quantitative* data, *and we want to explore more data qualitatively.*

*7-Male*.

Participants understood that they required a strong justification and a need to implement a mixed methods approach to answer their research questions. Some believed that when quantitative data were not sufficient to explain a certain phenomenon or construct, qualitative data can add to it. They believed that adding a qualitative component offered a better understanding of the answers to their research questions particularly inquiring about participants’ perceptions, feelings, and reflections about a particular topic. Others argued that using a mixed methods approach gives a strong backbone to the findings from the studies, especially when investigating a new concept. Others simply believed that conducting mixed methods research would increase their chances of publishing their research in higher ranked journals. Moreover, the participants were also aware that they could not use a mixed methods approach for just any type of research question, as this carries its own limitations in conducting research, including the factors of time, resources, target population, and settings.

*Because in medical education, we cannot quantify everything in numbers since medical education has many aspects that have perceptions, emotions, feelings, and reflections to generalize it. 14-Female*.

*I also found that the mixed methods methodology is frequently used in medical education research and publications especially when you are studying a phenomenon and you want to see the impact of that phenomena. 12-Female*.

*It depends on research question, we shouldn’t use mixed methods methodology just because of the availability of both quantitative and qualitative data. 15-Male*.

### Theme 2: strategies and integration in mixed methods implementation

When inquired about if the participants clearly stated the objectives or the research questions being addressed by the mixed methods research, they were divided in their responses, where some clearly identified and stated their objectives while others did not.

*I had three research questions to address the aim of the study, a quantitative, a qualitative and a mixed method methodology question. 12-Female*.

*One question is sufficient in mixed methods methodology to be answered by the quantitative and qualitative study. 14-Female*.

Participants were sometimes advised to explore their research questions in more detail, at times more complex than what could be answered through a simplistic design, leading them to consider a mixed-methods research design. Some explained how they searched for literature on their topics, and from there they were inspired to employ a mixed methods design. A participant explained that a mixed methods approach was necessary in her case because she believed it added value to the reliability, validity, and generalizability of the data. Other participants used mixed methods research because they needed to investigate patterns in their samples that quantitative analysis alone could not answer, such as perceptions about a specific phenomenon.

*It depends on research question, we shouldn’t use mixed methods methodology just because of the availability of both quantitative and qualitative data. 15-Male*.

*(I added) a qualitative study to get more in-depth data and which will explain what we are going to find in the quantitative data. I also started reading similar studies to see which method is best for such studies, and I found out that mixed methods methodology was the most suitable one. 12-Female*.

*I usually use mixed methods methodology studies when I need to explore perceptions, experiences, and challenges because first I must identify these challenges and experiences and then further explore them in-depth. 5-Female*.

For most participants, the mixed methods approach was driven by the research questions that they formulated at the outset of planning their research. For some, it was part of the theoretical frameworks they wanted to use in their research, requiring a comprehensive approach. Others just found at a later stage of their research, to employ a mixed methods approach, whereas for others, the available data required them to use this approach.

*So, my research questions in the qualitative part were guided by these components, like assignments, clinical evaluations, and exams. 12-Female*.

*So, it depends on the cognitive or theoretical framework that serves your purpose. 10-Male*.

Most participants in our study acknowledged the need for clarity about using an appropriate study design at the outset. A participant said that if the design was wrong at the beginning, it would lead to poor data and half-answered research questions. Participants talked about considering how the data sets would be analyzed in a mixed methods design. They were particularly aware of the need for integration of the findings in the discussion part of their report.

*If you have good quantitative and qualitative parts in your study, that doesn’t mean you will be able to mix the results together for a mixed methods methodology study. 10-Male*.

*The merging of results will also occur when I discuss the results in the discussion part of the study. 15-Male*.

When asked about how they prioritized a component in their mixed methods design, participants talked about the way they selected the components. Most believed that both the components supported each other, but they had to consider the factors that could affect the decision about which part of the study could be started first, including the factors of feasibility and resource availability, starting with the quantitative to address the research questions. However, for studies for which participants were required to implement an exploratory design, they mostly started with a qualitative design followed by a quantitative one, which was supported by items from the preliminary analysis of qualitative data. However, a participant said that explanatory designs start with quantitative rather than qualitative designs. Some participants who did not employ a sequential procedure used both components simultaneously. Nevertheless, the participants were aware of the logical sequence of the components of the mixed methods research.

*It is very important to prioritize data sets to select the appropriate design. 13- Male*.

*We still need to determine the time of data collection of one data set over the other even if the data sets have equal priority. 10-Male*.

*Despite of weather the design is sequential or concurrent, you must decide which data set you will start with, quantitative or qualitative and must give the rationale behind it. 6-Male*.

*We can prioritize the data in triangulation based on the phenomena that we have and what the relevant data and resources are that we will explore. 5-Female*.

*If they are not depending on each other then I will see other factors that could affect which one can be started first, such as feasibility and available resources to start with the quantitative to answer the first or second objective then I will do this. 1-Female*.

Integration and making sense of the data originating from two different sources of data was another major challenge for the study participants. They talked about situations where they attempted to reduce the data, but simultaneously, they were afraid of losing the richness in their data, which they believed was essential to capture the nuances in the data. A participant talked about the importance of merging by saying that it must be considered at the outset when the data is being collected and later when analysis begins. Nevertheless, most participants, except some, valued that merging was one of the most important aspects of mixed-method studies, as it added more weight and credibility to the study. They were able to give examples of how they merged their data in the discussion sections of their reports. However, many participants believed that their understanding of the methods of merging in such studies was limited; this is the reason, for instance, that a participant explained that he drafted the findings from two sets of data separately within a single report without performing merging of results.

*Then I will report it in the discussion part, the relation between both results. 12-Female*.

*The quantitative and qualitative studies should be linked together and merged to get the mixed methods methodology results. 10-Male*.

*We didn’t merge the quantitative and qualitative results. I kept them separately. 7-Male*.

*I stated the quantitative and qualitative results independently. 4-Male*.

*Data will be integrated in the results and in the discussion parts.*

*The study should have quantitative results, qualitative results and mixed methods methodology results. 12-Female*.

*I integrated the data in the results part and these merged results were discussed in the discussion part. 3-Female*.

*I had no specific technique to present the MMM results. 2-Male*.

*Yes. I did a table that shows my results for qualitative and quantitative studies and the* mixed methods methodology *results. 3-Female*.

*I displayed my* mixed methods methodology *results using tables. I had to interpret some words into numbers.*

*4-Male*.

*Technique to merge data depends on the design used and depends on the researcher. 5-Female*.

### Theme 3: mastery in mixed methods: prerequisites and expert consultation in research

The participants talked about several issues pertaining to the prerequisites for conducting a mixed methods study. Experience in qualitative research techniques was valued as crucial prerequisites for a researcher’s readiness to use a mixed methods research approach. For the participants, resources, and feasibility, apart from a strong rationale, some experience, and knowledge, were one of the main prerequisites for conducting the qualitative component of the mixed methods research.

*The qualitative section specifies the type of qualitative method you will employ focus groups, interviews, formal or informal interviews, and whether you have sufficient manpower resources to conduct this interview. 1-Female*.

*You must have experience of conducting both quantitative and qualitative studies and good exposure to have a well-structured mixed methods methodology study. 5-Female*.

Participants also talked about how using mixed methods research was a challenge for them and how they upgraded their skills to accomplish their complex projects. It required some to do extra reading before they initiated their projects. For many, it meant getting upskilled in qualitative research methods, as they already had adequate training in quantitative skills. In doing so, they believed that access to resources and resourceful people who could impart qualitative skills in their institutions was essential to their projects. Some participants, therefore, indicated the need for formal training in mixed methods research and qualitative techniques. This is because they believed that these types of studies needed to be designed perfectly, since if the design is wrong at the beginning, the data may not be rich enough to answer the research questions.

*You must have enough knowledge about the mixed methods methodology studies before doing one. 5-Female*.

*I personally took workshops in conducting qualitative studies because I only had a background in quantitative studies. 3-Female*.

*You must have training in mixed methods methodology and exposure to conduct mixed studies. 5-Female*.

*It is very important to have an expert in mixed methods methodology even if we have experienced researchers in quantitative and qualitative studies. 13-Male*.

Participants discussed how important it was to consult an expert with a mixed research methods background, especially at the start. They believed it would enhance the quality of the study because, even if the researcher has a good background in both quantitative and qualitative studies, they still required guidance throughout the study in analysis, writing results, and discussion because it is different than quantitative and qualitative studies. The participants insisted that even if a researcher has a background in both quantitative and qualitative research, it is always better to consult an expert with experience in mixed methods research.

*It is very important to have an expert in the field of mixed methods methodology because he will be extremely helpful in integrating the data together. 15-Male*.

*I think we need experts in mixed methods methodology, even if the researcher has a good background in both quantitative and qualitative studies because you will need them to guide you through the study in analysis part, writing results and discussion parts because it is different than quantitative and qualitative studies. 12-Female*.

They did, however, believe that it was difficult to find qualitative researchers who were knowledgeable about advising on mixed methods research. They argued that the supervisors should be aware of the mixed methods design so that they are able to advise on the appropriateness of conducting studies with advanced study designs. Some believed that, ideally, the researcher should have a supervisor with a sound background in mixed methods research because it is not enough that the researcher has either a background in quantitative or qualitative research alone. Whereas others thought that specialist mixed methods researchers may not be needed in situations where a person who is expert equally in quantitative and qualitative approaches.

*It is better to have an expert in mixed methods methodology look at the themes and see how you will analyze your data. 3-Female*.

*We don’t need to involve a mixed methods methodology expert while doing the research if we have researchers with good experience in both quantitative and qualitative studies. 14-Female*.

## Discussion

Our study’s findings indicate a diverse range of understandings of mixed methods methodology among medical education researchers. Some of the participants understood mixed methods methodology, when it should be used, and how it should be implemented. However, the responses from several of the participants indicated a lack of understanding about mixed methods methodology utilization. From the responses provided by the participants, three important issues were identified that need to be discussed. These issues are related to how mixed methods methodology is defined, the reason to use mixed methods methodology as opposed to only using a qualitative or quantitative methodology, and the process of presenting the results of a methods study. Table 2 lists the problems identified in the data analyzed from the participants. Each of these issues are important because of the indication that some of the participants did not fully understand mixed methods methodology and how to select and use the methodology to achieve the best outcomes for medical research.

**Table 2:** Problems With Mixed Methods Methodology Identified in the Study

• Lack of understanding of the definition of mixed methods methodology
• Not understanding when to use mixed methods methodology over other methodologies
• Not understanding how to achieve integration in mixed methods methodology
• Presenting qualitative and quantitative aspects of mixed methods independently

### Defining mixed methods methodology

The simplistic definitions and explanations provided by some participants highlight a fundamental challenge. Some of the participants defined mixed methods methodology as simply being the combined use of both qualitative and quantitative methods in a single research study. In this regard, the participants understood the composition of mixed methods methodology, but did not have a more in-depth understanding that mixed methods methodology is about bringing together the inductive nature of a qualitative methodology and the deductive nature of a quantitative methodology to examine a research problem more broadly [9].

Furthermore, the participants did not explain that mixed methods methodology can be treated as a distinctive methodology on its own separate from qualitative and quantitative methodologies [10]. Instead, the participants perceived that the mixed methods methodology is solely about bringing qualitative and quantitative methods together. However, some of the participants stated that by bringing both qualitative and quantitative components together, it was possible to gain a more in-depth understanding of the feelings or perceptions of the people being studied.

Some of the participants also believed that conducting a mixed-methods study involved combining qualitative and quantitative data during the analysis. From this perspective, the mixed methods methodology was not viewed as a specific methodology that guided all the work that was performed from formulating research questions to determining the best methodology to use to answer those questions [8]. Instead, a mixed methods approach was only thought of as something to be considered when engaging in data analysis.

One other aspect of the responses provided by the participants about how they defined and perceived the mixed methods methodology was that some of the participants seemed to view this methodology as a means of getting published in higher ranking journals. Some of the participants stated that they recognized that mixed methods studies were more desirable for some journals because of the ability to gain broader and more in-depth data for analysis. In this way, the use of mixed methods was perceived to be a utility to increase the potential to have studies published in more respectable journals rather than as a distinct research methodology.

### Appropriately using mixed methods methodology

Another issue that was identified in the responses provided by the participants was that some of the participants did not understand the appropriate use of mixed methods methodology. As has already been discussed, some of the participants believed that mixed methods methodology should be used to increase the potential of having a study published in a higher-ranking journal. However, other participants described using mixed methods methodology to gain a better understanding of the perceptions and feelings of participants. While mixed methods methodology can be used to gain a more in-depth understanding of a problem, it is not always appropriate to use this methodology even if the goal is to understand the perceptions or feelings of participants.

Instead, the use of qualitative methods can allow a researcher to gain data about the perceptions and beliefs of participants in relation to a problem or phenomenon. Using a qualitative methodology over mixed methods methodology is more appropriate in many circumstances when the goal is to understand the perceptions and beliefs of a group of individuals. The mixed methods methodology is not appropriate in all situations in which the goal is to gain data about the perceptions of participants. In this regard, there was a lack of understanding among some of the participants about when it is appropriate to use a qualitative methodology and when it is appropriate to use mixed methods methodology.

An important aspect of the misunderstanding among some of the participants about when to use mixed methods methodology was that the mixed methods methodology should be used when the problem being investigated and the type of data that are needed to address a research problem warrant its use. One of the participants stated that mixed methods methodology was appropriate when investigating a new concept. The investigation of a new concept might warrant the use of mixed methods methodology, but the use of a qualitative methodology or a quantitative methodology may be more appropriate depending on the research problem being examined and the goals of the study.

### Presenting the results of a mixed methods study

The third issue that was identified in the responses provided by the participants was about how the results of a mixed methods study should be presented. The participants generally explained that the results of the qualitative and quantitative portions of a mixed methods study should be presented independently of each other. This is problematic because the participants viewed the qualitative and quantitative aspects of a mixed methods study to be independent of each other rather than as being used together to gain a deeper understanding of a research problem. There was a lack of understanding that in a mixed methods study, the qualitative and quantitative data that are collected should be merged and presented together in a way that answered the research questions for which the data were collected [23].

On a broader level, the idea that the qualitative and quantitative data in a mixed methods study would be presented independently of each other leads back to the issue of correctly using the mixed methods methodology as part of the design of a study. A mixed methods study should be conducted so that the qualitative and quantitative data are used together to address the research problem being investigated. The qualitative and quantitative data that are collected should not be treated as being independent of each other. If the qualitative and quantitative data are independent of each other, then mixed methods methodology has not been fully used to gain an in-depth understanding of an issue or phenomenon [2]. Instead, two research methods were used and brought together separately.

Finally, a mixed methods study does not require that half of the data presentation be used to present the quantitative data results and half of the presentation be used to present the qualitative data results. Instead, the data should be presented together in a way that is appropriate for the study that was conducted. In some studies, this might mean that more qualitative data are presented or that more quantitative data are presented. The important issue is to merge the qualitative and quantitative data and present them as one complete unit [9]. The lack of understanding among the participants about how to appropriately present the results of a mixed methods study also demonstrates that while some of the participants may have understood the idea of mixed methods methodology, they lacked a complete understanding of how to carry out a mixed methods study in terms of presenting the data. In this regard, the participants did not demonstrate a full understanding of the entire process of conducting research using the mixed methods methodology.

Based on the methods used to conduct this study and the findings of the study, several recommendations can be made both for future research and for practice in medical education. One of the recommendations for medical education practice is that training needs to be provided in medical education about mixed methods methodology. While training in mixed methods methodology is common in other disciplines, it is still relatively new in medical education. Increasing the training that students receive about mixed methods methodology would help to increase the general knowledge that exists of the methodology within medical education and its use among medical researchers.

Another recommendation for medical education is to change how research methodologies are presented to students. In medical education, it is still common for quantitative research to be presented as more appropriate for medical research [6]. For the use of mixed methods methodology to increase within medicine, future researchers in medicine need to be trained that quantitative methodology is not the only appropriate methodology for medical studies. However, they also need to be trained that mixed methods methodology is one methodology that can be used, but it should not be used in all situations.

In terms of future research, one recommendation is for future research to be conducted on how mixed methods methodology is being used in published medical education research. It would be useful to examine if mixed methods methodology is being used appropriately in published medical studies. By examining published mixed methods studied in medical education, an examination could occur about how mixed methods methodology is used and whether it is used in a way that integrates qualitative and quantitative methods.

### Contribution to mixed methods research

The results of this study are useful as a contribution to mixed methods research because more has been learned about the perceptions and knowledge that exist about mixed methods among medical education faculty. Mixed methods methodology is still relatively underutilized as compared to quantitative methods within medical research and medical education. Based on the findings of this study, medical faculty lack a strong knowledge about how to implement mixed methods methodology and why it should be used as compared to other methodologies. The practical contribution that is made is that medical faculty need more training in mixed methods methodology, and future medical researchers and faculty need to receive more training in mixed methods methodology as part of their medical education.

## Conclusion

This study aimed to investigate the application and integration of mixed methods methodology in medical education research, with a focus on researchers’ perceptions, strategies, and readiness. The findings reveal a varied understanding of mixed methods among participants. While some researchers could articulate basic definitions, their comprehension often did not extend to a strategic or integrated use throughout the research process. This suggests a gap between recognizing mixed methods as a concept and effectively applying it in practice, encompassing both qualitative and quantitative approaches cohesively.

Researchers generally acknowledged the importance of mixed methods in medical education research but displayed a limited grasp of how to strategically implement and integrate these methods. This indicates a need for enhanced educational strategies in medical education to foster a more profound understanding and skillful application of mixed methods research.

The study highlights that readiness in terms of skills and expertise for conducting mixed methods research is not yet fully developed among medical education researchers. There is a growing interest in mixed methods, but it is often seen as a means to an end, such as achieving publication, rather than as a comprehensive methodology suited to certain types of research questions.

The limitation of the study lies in its small and non-representative sample, which challenges the generalizability of the findings. The focus was mainly on perceptions rather than the actual execution of mixed methods research. Future research should thus explore how medical education researchers apply mixed methods in real-world research settings, assessing the alignment between their theoretical understanding and practical application.

These findings are crucial for understanding the current state of mixed methods research in medical education and point towards the need for more targeted training and resources. By bridging the gap between theoretical knowledge and practical application, the quality and efficacy of mixed methods research in medical education can be significantly enhanced.

## Data Availability

The datasets used and/or analyzed during the current study are available from the corresponding author on responsible request.
